# Effect of angiotensin-converting enzyme gene I/D polymorphism and its expression on clinical outcome in acute respiratory distress syndrome

**DOI:** 10.1186/cc12040

**Published:** 2013-03-19

**Authors:** I Tsangaris, A Tsantes, P Kopterides, G Tsaknis, S Kokkori, I Karampela, D Konstantonis, S Karabi, E Vrigkou, A Pappas, S Orfanos, A Armaganidis

**Affiliations:** 1 University Hospital 'Attikon', University of Athens Medical School, Athens, Greece

## Introduction

The role of the D allele of the angiotensin-converting enzyme (ACE) gene I/D polymorphism in the clinical outcomes of patients with acute lung injury and acute respiratory distress syndrome (ALI/ARDS) remains controversial. We assessed simultaneously the effect of the ACE I/D polymorphisms as well as the serum and BALF ACE levels on prognosis of ARDS patients.

## Methods

We recruited 69 mechanically ventilated ALI/ARDS patients. ACE activity levels in serum and BALF were assessed by chemical methods. Patients were genotyped for ACE I/D polymorphisms. Time-to-event analysis evaluated the variables associated with the 28-day and 90-day mortality.

## Results

**Figure 1:**
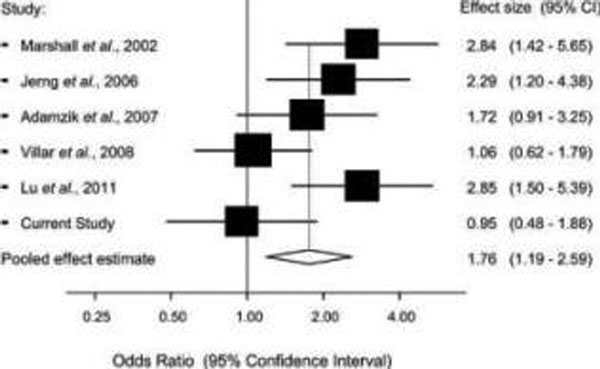


In the multivariable model, age, lung compliance, serum lactate and serum ACE levels were significantly associated with both 28-day and 90-day mortality. No significant correlation was found between serum and BALF ACE levels (Spearman's ρ = 0.054; *P *= 0.66). Serum ACE concentrations were significantly higher (*P *= 0.046) in patients with D/D genotype versus the two other groups combined (I/D and I/I genotypes). A meta-analysis of six studies (including ours) provided evidence that the D allele is significantly associated with increased mortality in ALI/ARDS patients, yielding a per-allele odds ratio of 1.76 (95% CI: 1.19 to 2.59). See Figure 1 and Table 1.

**Table 1:** Characteristics of the study population (*n *= 69)

Age (years)	64.4 ± 17.9; 69 (28 to 89)
Sex (males, %)	43 (62.3%)
APACHE II score	22.1 ± 6.2; 21 (14 to 35)
SOFA score	8.4 ± 3.2; 8 (4 to 16)
Lung compliance (ml/cmH_2_O)	29.4 ± 7.2; 30 (17 to 40)
PaO_2_/FiO_2_	139, 1 ± 47.1; 140 (72 to 223)
PEEP	8.1 ± 2.9; 8 (5 to 15)
Lung Injury Score	2.5 ± 0.6; 2.5 (1.75 to 3.50)
Blood lactate (mmol/l)	1.7 ± 1.7; 1.3 (0.5 to 5.2)
Septic status	39 (56.5%)
Serum ACE (U/l)	16.5 ± 10.8; 13.6 (4.8 to 39.8)
BALF (U/l)	2.3 ± 1.4; 2.0 (0.3 to 4.7)
ACE I/D polymorphism:	
*D/D*	27 (39.1%)
*I/D*	28 (40.6%)
*I/I*	14 (20.3%)
28-day mortality	34/69 (49.3%)
90-day mortality	43/69 (62.3%)
Ventilator-free days	5.1 ± 7.8; (0 to 23)
Days w/o cardiovascular failure	14.0 ± 10.2; 16 (0 to 27)
Days w/o renal failure	15.4 ± 10.6; 16 (0 to 28)

## Conclusion

Serum ACE levels appear to be affected by the I/D polymorphism and are correlated with prognosis in patients with ALI/ARDS, indicating that further investigation of the clinical significance of the ACE in ARDS might be of value.

